# Predicting *β*-turns and their types using predicted backbone dihedral angles and secondary structures

**DOI:** 10.1186/1471-2105-11-407

**Published:** 2010-07-31

**Authors:** Petros Kountouris, Jonathan D Hirst

**Affiliations:** 1 School of Chemistry, University of Nottingham, University Park, Nottingham NG7 2RD, UK

## Abstract

**Background:**

*β*-turns are secondary structure elements usually classified as coil. Their prediction is important, because of their role in protein folding and their frequent occurrence in protein chains.

**Results:**

We have developed a novel method that predicts *β*-turns and their types using information from multiple sequence alignments, predicted secondary structures and, for the first time, predicted dihedral angles. Our method uses support vector machines, a supervised classification technique, and is trained and tested on three established datasets of 426, 547 and 823 protein chains. We achieve a Matthews correlation coefficient of up to 0.49, when predicting the location of *β*-turns, the highest reported value to date. Moreover, the additional dihedral information improves the prediction of *β*-turn types I, II, IV, VIII and "non-specific", achieving correlation coefficients up to 0.39, 0.33, 0.27, 0.14 and 0.38, respectively. Our results are more accurate than other methods.

**Conclusions:**

We have created an accurate predictor of *β*-turns and their types. Our method, called DEBT, is available online at http://comp.chem.nottingham.ac.uk/debt/.

## Background

Secondary structure can provide important information about three-dimensional protein structure. Therefore, its prediction has been an area of intense research over the past three decades. To predict secondary structure many methods have been implemented, including different machine learning techniques, such as artificial neural networks (ANNs) [1,2] and support vector machines (SVMs) [3-5], and different input schemes, such as position specific scoring matrices (PSSMs) [2] and hidden Markov models [6]. Notably, the predictive accuracy reached 80% for three-state prediction, where residues are divided into helix, strand and coil. Helices and strands are repetitive, regular structures, while the remaining residues, which can be tight turns, loops, bulges or random coil, are all classified as coil; they are non-repetitive, irregular secondary structures [7]. Although the helix and strand classes are structurally well-defined, the third class, coil, does not provide any detailed structural information. Hence, further analysis of the local structure is necessary, such as prediction of backbone dihedral angles [5,8] and prediction of tight turns [9].

Tight turns play an important role in protein folding and stability. They are partly responsible for the compact, globular shape of proteins, because they provide directional change to the polypeptide chain [10]. Depending on the number of constituent residues, tight turns can be classified as *α*-turns, *β*-turns, *γ*-turns, *δ*-turns or *π*-turns. A *β*-turn is formed by four adjacent residues, which are not in an *α*-helix, where the distance between C_*a*_(*i*) and C_*a*_(*i *+ 3) is less than 7 Å [9]. The *β*-turns are the most common tight turns. On average, about a quarter of all residues are in a *β*-turn [11]. Moreover, *β*-turns are crucial components of *β*-hairpins, the fundamental elements of anti-parallel *β*-sheets, whose prediction has recently attracted interest [12-14]. Furthermore, *β*-turn formation is an important step in protein folding [15], while improved *β*-turn sequences can improve protein stability [16,17]. Additionally, their occurrence on the surface of proteins suggests their involvement in molecular recognition processes and their interactions between peptide substrates and receptors [18]. Recently, mimicking *β*-turns for the synthesis of medicines [19,20] or for nucleating *β*-sheet folding [21] has also attracted interest. Thus, the prediction of *β*-turns can facilitate three-dimensional structure prediction and can provide important information about the protein folding. Hutchinson and Thornton [22] classified the *β*-turns into nine types based on the dihedral angles of residues *i *+ 1 and *i *+ 2 in the turn (table 1). This is the most established classification of *β*-turns.

**Table 1:** The dihedral angles of *β*-turn types [22]

Turn type	Dihedral angles (°)			
	***ϕ***_***i *+ 1**_	***ψ***_***i *+ 1**_	***ϕ***_***i *+ 2**_	***ψ***_***i *+ 2**_
I	-60	-30	-90	0
I'	60	30	90	0
II	-60	120	80	0
II'	60	-120	-80	0
IV	-61	10	-53	17
VIa1	-60	120	-90	0
Via2	-120	120	-60	0
VIb	-135	135	-175	160
VIII	-60	-30	-120	120

*β*-turns are divided into nine classes based on the dihedral angles of the central residues. Type IV is a miscellaneous category that contains all conformations outside the other eight classes and the dihedral angles shown here are the average values and, therefore, depend on the dataset.

Prediction of *β*-turns has attracted interest in the past. The approaches can be divided into statistical methods and machine learning techniques. The former include early methods which used amino acid propensities [23-27] as well as more recent methods, like COUDES [28], which used probabilities with multiple sequence alignments. Over the past few years, machine learning techniques have been applied successfully to predict *β*-turns. Since their first use [29], ANNs have been frequently used for *β*-turn prediction [30-32]. Over the past decade, several studies used SVMs to predict *β*-turns [33-37] and other techniques, such as nearest neighbour, have been applied recently [38]. Through the use of evolutionary information and more sophisticated machine learning techniques, the correlation coefficient for turn/non-turn prediction is now as high as 0.47 [34]. Other methods predict the type of *β*-turn, rather than the location of the turn in the chain, with significant success, even though this problem is challenging, due to the lack of examples for many *β*-turn types. BTPRED [30], BetaTurns [39], MOLEBRNN [32] and the method of Asgary and colleagues [40] are ANN-based, whereas COUDES [28] uses amino acid propensities with multiple sequence alignments. In spite of its successful use for the prediction of *β*-turn location [34,37], the SVM method has not been employed widely for *β*-turn type prediction.

Despite the success so far, there is a need for more accurate predictions of both *β*-turn location and *β*-type, which could be realised through the use of additional information. Evolutionary information from multiple alignments [31] as well as predicted secondary structures [30] can improve *β*-turn predictions dramatically. In this work, we show that the backbone dihedral angles can provide crucial information for turn/non-turn prediction and can also noticeably improve the prediction of *β*-turn types, since the types are defined by the dihedral angles of the central residues. Predicted dihedral angles have been used successfully for secondary structure prediction [5,41]. The method presented here, called DEBT (Dihedrally Enhanced Beta Turn prediction), uses predicted secondary structures and predicted dihedral angles from DISSPred [5] and achieves the highest correlation coefficient reported to date for turn/non-turn prediction, while the prediction of *β*-turn types is, in most cases, more accurate than other contemporary methods. The method predicts *β*-turn type I, II, IV, VIII as defined by Hutchinson and Thornton [22], while all remaining types are classified as NS (non-specific). Moreover, we show that using a small local window of predicted secondary structures and dihedral angles, rather than using the predictions of one individual residue, is beneficial.

## Methods

### Datasets

DEBT was trained and tested on four non-redundant datasets, which contain chains with at least one *β*-turn and have X-ray crystallographic resolution better than 2.0 Å. All protein chains have less than 25% sequence similarity, to ensure that there is very little homology in the training set. The first dataset, denoted as GR426 in this paper, consists of 426 protein chains [42] and was used to study the impact of various training schemes and to tune the kernel parameters. GR426 has been used by the majority of recent *β*-turn prediction methods and, therefore, we can use it to make direct comparisons. In 2002, the GR426 dataset was used for the evaluation of *β*-turn prediction methods [43] and was partitioned into seven subsets in order to perform seven-fold cross-validation. In this work, we utilised the same partition for the cross-validation. After finding the optimal input scheme and tuning the kernel parameters, we used two additional datasets, constructed for training and testing COUDES [28], to validate the performance of our method. The datasets consist of 547 and 823 protein chains and are denoted as FA547 and FA823, respectively. Finally, DEBT's web-server was trained using PDB-Select25 (version October 2008) [44], a set of 4018 chains from the PDB with less than 25% sequence similarity. From these chains, we have selected those that contain at least one *β*-turn and have an X-ray crystallographic resolution below 2.0 Å. This gave a dataset of 1296 protein chains, denoted as PDB1296 in this article, which is the largest training set used for a *β*-turn prediction server. The PDB codes and chain identifiers of the above datasets are listed at DEBT's website http://comp.chem.nottingham.ac.uk/debt/. The *β*-turns and their types were assigned using the PROMOTIF program [45]. In this work, we predict *β*-turn types I, II, IV, VIII, while all the remaining types are assigned to the miscellaneous class NS (non-specific). Table 2 shows the distributions of *β*-turns and their types in each dataset.

**Table 2:** Distribution of residues in *β*-turns and their types in different datasets

*β*-turn types						
Dataset	*β*-turns (%)	I (%)	II (%)	IV (%)	VIII (%)	NS (%)
GR426	23.5	9.3	3.8	9.4	2.7	2.4
FA547	23.1	9.1	3.7	9.1	2.8	2.5
FA823	22.0	8.9	3.6	8.8	2.5	2.6
PDB1296	21.0	8.9	3.4	8.2	2.5	2.7

DEBT method utilises PSSMs, constructed by the PSI-BLAST algorithm [46], to predict *β*-turns and their types. PSSMs have *N *× 20 elements, where the *N *rows correspond to the length of the amino acid sequence and the columns correspond to the 20 standard amino acids. PSSMs represent the log-likelihood of a particular residue substitution, usually based on a weighted average of BLOSUM62 [47]. We generated the PSSMs using the BLOSUM62 substitution matrix with an E-value of 0.001 and three iterations against a non-reduntant (nr) database, which was downloaded in February 2009. The data were filtered by *pfilt *[48] to remove low complexity regions, transmembrane spans and coiled coil regions. The PSSM values were linearly scaled simply by dividing them by ten. Typically, PSSM values are in the range [-7,7], but some values outside this range may appear. Linear scaling maintains the same distribution in the input data and helps avoid numerical difficulties during training.

### Support Vector Machines

DEBT employs SVM [49], a state-of-the-art supervised learning technique. The SVM method has become an area of intense research, because it performs well with real-world problems, it is simple to understand and implement and, most importantly, it finds the global solution, while other methods, like ANNs, have several local solutions [50]. The SVM can find non-linear boundaries between two classes by using a kernel function, which maps the data from the input space into a richer feature space, where linear boundaries can be implemented. Furthermore, the SVM effectively handles large feature spaces, since it does not suffer from the "curse of dimensionality", and, therefore, avoids overfitting, a common drawback of supervised learning techniques.

A detailed description of the SVM algorithm can be found in various textbooks [50-52]. In brief, given input vectors **x_i _**∈ *R^n ^*and output values *y*_*i *_∈ {-1, 1}, the fundamental goal of a binary SVM classifier is to solve the following optimisation problem:(1)

where **w **is a vector perpendicular to the hyperplane, *b *is the offset from the origin and *C *is a penalty parameter for each misclassification. Thus, it controls the trade-off between training error and the margin that separates the two classes. The kernel function used in our case is the radial basis function (RBF), shown in equation 2, which was successfully used for complex problems, such as secondary structure prediction [3] and dihedral prediction [5].(2)

where **x_i _**and **x_j _**are the input vectors for instances *i *and *j*, respectively, and *γ *is a parameter that controls the width of the kernel.

LibSVM [53], a popular SVM software package, was employed for the training and testing of the SVM classifiers. In order to get the optimal predictive performance, we optimised three parameters: C (equation 1), *γ *(equation 2) and *w*. The latter controls the cost of misclassification for the minority class and, therefore, reduces the effect of the imbalance in the datasets. In other words, different penalty parameters costs are used for each class [54]. The optimised parameters for each classifier are shown in table 3. Seven-fold cross-validation was applied on datasets GR426, FA547 and FA823. For the former, we utilised the the same subsets used by Kaur and Raghava [55] to evaluate different *β*-turn prediction methods, whereas the partition of the other two datasets was identical to the one used to train COUDES [28].

**Table 3:** Optimised parameters for each SVM classifier used in DEBT.

Classifier	*C*	*γ*	*w*
turn/non-turn	1	0.04	2
I/non-I	1	0.01	7
II/non-II	0.5	0.03	20
IV/non-IV	1	0.01	7.5
VIII/non-VIII	0.5	0.01	20
NS/non-NS	4	0.06	36

The parameters were optimised using the grid search approach.

### DEBT architecture

**Figure 1:**
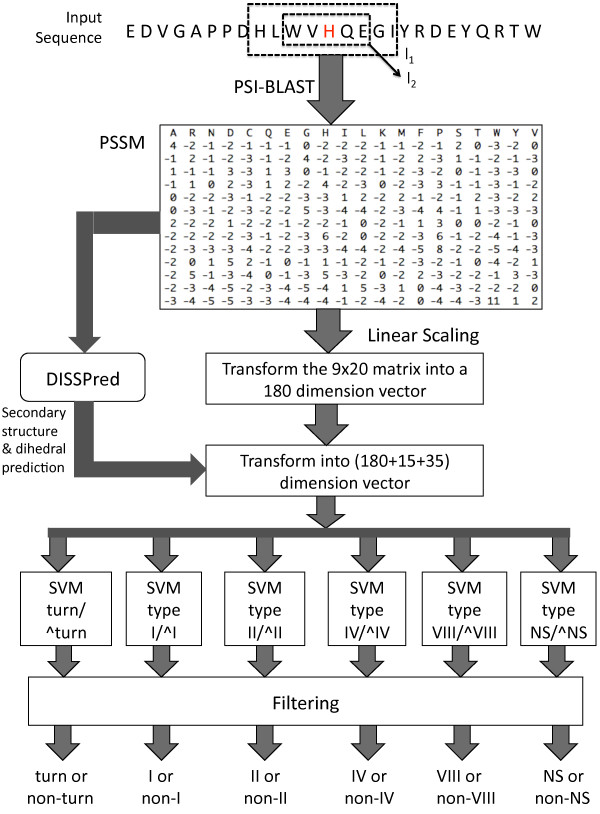
**The architecture of our *β*-turn location and *β*-turn type prediction method**. An example of an input sequence is provided at the top. Around each residue to be predicted (shown in red), two local windows are used. One, *l*_1_, has a size of nine residues and is used for the PSSM values, while the other, *l*_2_, takes in account the predicted secondary structures and dihedral angles for five residues. After running PSI-BLAST [46], the PSSM values are linearly scaled and transformed into a vector of 180 attributes (i.e. a local window of nine residues, *l*_1_). DISSPred [5] utilises PSSMs to predict three-state secondary structures and seven-state dihedral angles, which are transformed into a vector of 50 attributes using a window of five residues (*l*_2_). The two vectors are merged to create the final input vector for the SVM classifiers. Lastly, the predictions are filtered to give the final result.

Figure 1 shows the architecture of the method. DEBT uses two different local windows around the residue to be predicted: one, *l*_1_, of nine residues for the PSSM values and a second, *l*_2_, of five residues for the predicted secondary structures and dihedral angles, both centred around the residue to be predicted. DISSPred [5] is used to predict both three-state secondary structure and the dihedral angles. DISSPred uses different partitions of the *ϕ *- *ψ *space created by two unsupervised clustering algorithms and both the algorithm and the number of clusters can be adjusted by the user. Subsequently, DISSPred predicts the secondary structure and the dihedral angles using an iterative process. For each residue in window *l*_2_, the predicted secondary structures are encoded using three binary attributes, one for each state: (1,0,0) for helix, (0,1,0) for strand and (0,0,1) for coil. The dihedral angles are predicted by DISSPred using a partition of seven clusters and, therefore, are encoded similarly using seven binary attributes. Thus, the input vectors of the SVM classifiers have 230 attributes: 180 attributes for the PSSM values, 15 attributes for the predicted secondary structures and 35 attributes for the predicted dihedral clusters. We used the same architecture for both turn/non-turn prediction and *β*-turn type prediction.

### Filtering

Because the prediction is based on individual residues, the SVM outputs include some *β*-turns that are shorter than four residues, which is unrealistic. Turn predictions longer than four adjacent residues are acceptable, since there are many *β*-turns in the dataset that are overlapping. In fact, about 58% are multiple turns [22]. To ensure that the predictions are at least four residue long, we applied some filtering rules similar to the "state-flipping" rule described by Shepherd and colleagues [30]. The rules are applied with the following order: (1) flip isolated non-turn predictions to turn (tnt → ttt), (2) flip isolated turn predictions to non-turn (ntn → nnn), (3) flip isolated turn pairs of turn prediction to non-turn (nttn → nnnn) and (4) flip the adjacent non-turn predictions to turn for isolated three consecutive turn predictions (ntttn → ttttt).

### Prediction accuracy assessment

Six different scalar measures were used to assess DEBT's performance. All of them can be derived from two or more of the following quantities: (1) true positives, *p*_*i*_, is the number of correctly classified *β*-turns or *β*-turn type *i*, (2) true negatives, *n*_*i*_, is the number of correctly classified non-turns, (3) false positives, *o*_*i*_, is the number of non-turns incorrectly classified as *β*-turns or *β*-turn type *i *(over-predictions), (4) false negatives, *u*_*i*_, is the number of *β*-turns or *β*-turn type i incorrectly classified as non-turn (under-predictions) and (5) total number of residues, *t *= *p*_*i *_+ *n*_*i *_+ *o*_*i *_+ *u*_*i*_, where *i *= I, II, IV, VIII or NS. The first measure used is the predictive accuracy, the percentage of correctly classified residues.(3)

Two measures, that are usually used together, are sensitivity (also labelled as *Q*_*obs *_in some articles) and specificity which give the percentage of observed *β*-turns or *β*-turn types that are predicted correctly and the percentage of observed non-turns that are predicted correctly, respectively. The optimal is to equalise the two measures.(4)(5)

We report the commonly used Matthews correlation coefficient (MCC) [56], which is the most robust measure for *β*-turn prediction. The reason is that, when the dataset is unbalanced, it is possible to achieve high predictive accuracy just by predicting all instances as non-turn. The MCC, defined by equation 6, is a number between -1 and 1, with perfect correlation giving a coefficient equal to 1. Therefore, a higher MCC corresponds to a better predictive performance.(6)

Finally, we report *Q*_*pred*_, the percentage of *β*-turn predictions that are correct:(7)

Another important consideration is whether the classifiers perform better than random prediction. Herein, we report a normalised percentage better than random (*S*_*i*_), defined in equation 8, which was introduced by Shepherd and colleagues [30]. Perfect predictions score *S*_*i *_= 100%, whereas *S*_*i *_= 0% shows that the prediction is no better than random.(8)

where *R *is the expected number of residues that would be predicted correctly by a random prediction and is defined as:(9)

Apart from the scalar measures described above, we report the receive-operator characteristics (ROC) curves, which represent the sensitivity (or true positive rate - TP rate) against the false positive rate (1 - specificity). ROC curves have been widely used in bioinformatics [57] for visualisation and assessment of machine learning classifiers. Moreover, the area under the ROC curve (AUC) is calculated to provide a scalar measure of the ROC analysis and compare different methods. The trapezium rule is used to calculate the AUC, as described by Fawcett [58].

## Results and Discussion

### The effect of the input scheme

Before optimising the SVM classifiers, we tried different input schemes, which showed that the combination of evolutionary information (PSSMs), predicted secondary structures and predicted dihedral angles gives the most accurate predictions. Table 4 shows the results on the GR426 dataset from the experiments using various input schemes and different window sizes for the turn/non-turn classifier. Firstly, we changed the size of the PSSM window, *l*_1_, by using lengths of seven, nine and eleven residues. The last two sizes give the highest MCC value. We selected a window size of nine residues, because the input vector is smaller and, therefore, the training time is shorter. Subsequently, we augmented the PSSM-only input vector with additional attributes only for the central residue (i.e. *l*_2 _= 1) using predicted secondary structures, predicted dihedral angles or both. The results show that, when used together, predicted secondary structures and dihedral angles achieve the best performance. Finally, we changed the size of the second window, *l*_2_, using three, five or seven residues. The optimal window size is five residues. The same window sizes, *l_1 _*and *l*_2_, were utilised for all classifiers.

**Table 4:** Experiments on the GR426 dataset with different input schemes.

Input	***l***_**1**_	***l***_**2**_	MCC	Accuracy (%)
PSSM-only	7	0	0.369	69.7
PSSM-only	9	0	0.387	70.3
PSSM-only	11	0	0.387	69.9
PSSM + SS	9	1	0.404	72.2
PSSM + Dih	9	1	0.398	71.4
PSSM + SS + Dih	9	1	0.413	73.2
PSSM + SS + Dih	9	3	0.419	74.2
PSSM + SS + Dih	9	5	0.424	76.0
PSSM + SS + Dih	9	7	0.421	76.7

Window sizes, *l*_1 _and *l*_2_, are the windows for PSSM values and predicted secondary structures and/or dihedral angles, respectively. PSSM-only: only scaled PSSM values are used in the input vector; PSSM + SS: the input vector is augmented with predicted secondary structures; PSSM + Dih: the input vector is augmented with predicted dihedral angles; PSSM + SS + Dih: the input vector is augmented with both predicted secondary structures and predicted dihedral angles.

### Turn/non-turn prediction

Predicted dihedral angles and secondary structures improve the performance of the turn/non-turn classifier, as shown in table 5. In fact, the MCC shows an improvement of over 10% and reaches values of 0.48, 0.49 and 0.48 for datasets GR426, FA547 and FA823, respectively. Moreover, the overall accuracy is higher than 80% for datasets FA547 and FA823, while it is 79.2% for the GR426 dataset. Finally, *Q*_*pred*_, *Q*_*obs *_(sensitivity) and the better-than-random score, *S*, also improved after using predicted dihedral angles and secondary structures.

**Table 5:** Performance of DEBT for the prediction of *β*-turn location on three datasets.

Dataset	MCC	***Q***_***total ***_**(%)**	***Q***_***pred ***_**(%)**	***Q***_***obs ***_**(%)**	*S*	AUC
GR426	0.48 (0.43)	79.2 (78.6)	54.8 (53.9)	70.1 (61.6)	47.5 (43.2)	0.84 (0.83)
FA547	0.49 (0.44)	80.0 (79.2)	55.9 (54.5)	68.7 (60.5)	48.3 (43.6)	0.85 (0.83)
FA823	0.48 (0.42)	80.9 (79.9)	55.9 (54.1)	66.1 (56.5)	48.0 (42.3)	0.84 (0.82)

In the parentheses are the predictions using PSSM-only input. There is significant improvement on all measures when the input vector is augmented by predicted dihedral angles and secondary structures.

Table 6 compares the DEBT's predictive performance with other turn/non-turn predictors in the literature on the established datasets GR426, FA547 and FA823, sorted by the reported MCC score. The comparison is based on the MCC value, because it is the most robust measure, particularly when the dataset is unbalanced. Our achieved MCC values are the highest reported to date on all datasets. Interestingly, the methods by Zheng and Kurgan [34] and by Hu and Li [37], which report the second highest MCC score (0.47) on the GR426 dataset, are also SVM-based, which highlights the superiority of the SVM method compared to other machine learning techniques for *β*-turn prediction. Moreover, our method achieves a high MCC score by using a single SVM model, without any preprocessing, feature selection or predictions from multiple secondary structure or dihedral prediction methods, which may, potentially, improve the results. DEBT's performance using other measures is also one of the highest in the literature with overall accuracy around 80% and the *Q*_*pred *_and *Q*_*obs *_scores around 55% and 70%, respectively. These measures can vary depending on the balance of the dataset and the selected SVM parameters (table 3), which we optimised based on the more robust MCC score.

**Table 6:** Comparison of DEBT with other turn/non-turn prediction methods on three different datasets.

Dataset	*β*-turn predictor	MCC	***Q***_***total ***_**(%)**	***Q***_***pred ***_**(%)**	***Q***_***obs ***_**(%)**
GR426	DEBT	0.48	79.2	54.8	70.1
	Zheng and Kurgan [34]	0.47	80.9	62.7	55.6
	Hu and Li [37]	0.47	79.8	55.6	68.9
	Zhang et al. [35]	0.45	77.3	53.1	67.0
	BTSVM [36]	0.45	78.7	56.0	62.0
	MOLEBRNN [32]	0.45	77.9	53.9	66.0
	BETAPRED2 [31]	0.43	75.5	49.8	72.3
	COUDES [28]	0.42	74.8	48.8	69.9
	Kim [38]	0.40	75.0	46.5	66.7
	BTPRED [30]	0.35	74.4	48.3	57.3
					
FA547	DEBT	0.49	80.0	55.9	68.7
	Zheng and Kurgan [34]	0.45	80.5	61.6	54.2
	COUDES [28]	0.42	74.6	48.7	70.4
	Hu and Li [37]	0.43	76.6	47.6	70.2
					
FA823	DEBT	0.48	80.9	55.9	66.1
	Zheng and Kurgan [34]	0.45	80.6	60.8	54.6
	COUDES [28]	0.41	74.2	47.5	69.6
	Hu and Li [37]	0.45	76.8	53.0	72.3

The methods are sorted by their reported MCC score. DEBT achieves the highest value on all datasets.

### Prediction of *β*-turn types

Table 7 shows the performance of our method for the prediction of *β*-turn types on three different datasets. Notably, the MCC score increases dramatically when we augment the input vector with a local window of predicted dihedral angles and secondary structures. The improvement of the MCC score is at least 16%, 7%, 17%, 40% and 11% for types I, II, IV, VIII and NS, respectively, on all datasets. The explanation for the dramatic improvement of the prediction of some types, such as types I and VIII, can be derived from their dihedral angles (table 1). These types have negative *ϕ *and *ψ *angles and, hence, their structure is closer to a helical conformation, which is more accurately predicted by DISSPRED [5]. Therefore, more accurate secondary structure and dihedral predictions lead to more accurate *β*-turn type predictions. DEBT's predictive accuracy is over 70% for all types, with the caveat that it is not a reliable measure when the dataset is unbalanced. The prediction of the NS class with the highest MCC score clearly reflects the under-predictions, since the specificity is high and the sensitivity is low. When we attempted to equalise the two measures on the GR426 dataset, the MCC value dropped to 0.22, with the sensitivity and specificity at 68.5% and 84.3%, respectively. For all datasets, the better-than-random scores, *S*, are higher than 20% for all *β*-turn types except type VIII. On the GR426 dataset, DEBT's achieved *S *scores of 30.1%, 23.1%, 20.4% and 26.2% for types I, II, IV and NS, respectively, are noticeably higher than the scores reported by BTPRED [30] and BetaTurns [39]. The former achieved better-than-random scores of 18.1%, 18.9%, 4.5% and 2.6% for types I, II, VIII and IV, respectively, while BetaTurns reported values of 19.1%, 23.2%, 12.4%, 1.8% and 6.1% for types I, II, IV, VIII and NS, respectively.

**Table 7:** DEBT's prediction of *β*-turn types on three different datasets.

Dataset	*β*-turn type	MCC	Sensitivity (%)	Specificity (%)	***Q***_***total ***_**(%)**	*S *(%)	AUC
GR426	I	0.36 (0.31)	75.2 (67.5)	78.9 (78.4)	78.6 (77.9)	30.1 (26.2)	0.85 (0.82)
	II	0.29 (0.27)	63.4 (65.0)	88.3 (86.4)	87.4 (85.7)	23.1 (20.6)	0.87 (0.86)
	IV	0.27 (0.23)	71.2 (63.4)	71.5 (73.5)	71.5 (72.5)	20.4 (18.5)	0.78 (0.76)
	VIII	0.14 (0.10)	68.7 (29.1)	71.1 (89.8)	71.1 (88.1)	8.0 (7.7)	0.77 (0.73)
	NS	0.31 (0.28)	18.0 (19.8)	99.7 (99.4)	97.6 (97.4)	26.5 (26.1)	0.81 (0.81)
							
FA547	I	0.38 (0.31)	71.6 (66.6)	82.6 (79.5)	81.6 (78.3)	33.0 (26.0)	0.85 (0.82)
	II	0.33 (0.27)	63.0 (64.9)	90.8 (86.8)	89.8 (85.9)	27.8 (20.9)	0.88 (0.86)
	IV	0.27 (0.24)	69.8 (61.3)	73.3 (75.6)	73.0 (74.3)	21.0 (19.2)	0.79 (0.77)
	VIII	0.14 (0.10)	47.8 (28.4)	84.4 (90.2)	83.4 (88.5)	9.5 (7.9)	0.77 (0.73)
	NS	0.37 (0.28)	21.1 (21.2)	99.7 (99.2)	97.7 (97.2)	31.2 (26.3)	0.84 (0.82)
							
FA823	I	0.39 (0.30)	70.6 (64.3)	84.2 (80.7)	83.0 (79.3)	34.5 (26.0)	0.86 (0.82)
	II	0.33 (0.28)	62.7 (65.1)	91.2 (87.2)	90.2 (86.4)	27.9 (21.1)	0.88 (0.86)
	IV	0.27 (0.23)	68.3 (58.6)	74.4 (77.1)	73.9 (75.5)	21.0 (18.9)	0.79 (0.76)
	VIII	0.14 (0.08)	42.2 (12.4)	87.2 (96.6)	86.1 (94.5)	9.4 (7.3)	0.77 (0.72)
	NS	0.38 (0.29)	23.6 (24.2)	99.7 (98.9)	97.7 (97.0)	33.9 (27.9)	0.85 (0.83)

In the parentheses is the prediction using PSSM-only input without predicted dihedral angles or secondary structure. Notably, there is improvement in the predictive performance when the input vector is augmented by predicted dihedral angles and secondary structures.

Table 8 compares the performance of *β*-turn prediction with other methods in the literature based on the GR426 dataset. DEBT outperforms other contemporary methods for the prediction of type I, IV, VIII and NS. Our achieved MCC score is higher by at least 12.5% for types I and IV and by at least 27% and 29% for types VIII and NS, respectively. The performance highlights the importance of predicted dihedral angles in *β*-turn type prediction, since they are defined by the dihedral angles of the central residues (table 1). The prediction of type II is the only one that does not achieve a MCC score as high as some other methods. MOLEBRNN [32] and *- *using different dataset *- *the method by Asgary and co-workers [40] report higher MCC values, while COUDES [28] reports an MCC of 0.30, which is slightly higher than our achieved value of 0.29. However, DEBT achieves a comparable MCC of 0.33 for the prediction of type II using datasets FA547 and FA823, which generally give higher MCC values than GR426 for *β*-turn type prediction (see table 7).

**Table 8:** Performance of DEBT and other *β*-turn type prediction methods based on the achieved MCC value.

Prediction method	MCC score for each *β*-turn type				
	I	II	IV	VIII	NS
DEBT	0.36	0.29	0.27	0.14	0.31
BETATURNS [39]	0.22	0.24	0.16	0.02	0.05
COUDES [28]	0.31	0.30	0.11	0.07	--
MOLEBRNN [32]	0.32	0.34	0.24	0.11	--
BTPRED [30]	0.22	0.25	0.06	0.03	--
Asgary et al. [40]	0.24	0.47	0.10	0.12	0.24

DEBT is more accurate that other methods in the prediction of types I, IV, VIII and NS. The results for BTPRED and the method by Asgary and colleagues are obtained using different datasets.

### ROC analysis

**Figure 2:**
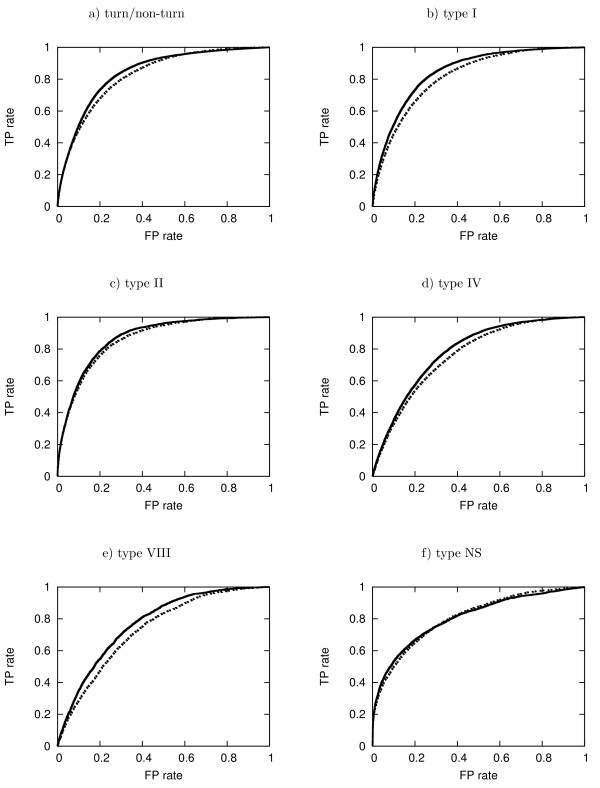
**ROC curves for the prediction on the GR426 dataset**. Dashed curves correspond to the PSSM-only prediction, while solid curves correspond to the prediction after augmenting the input vector with predicted dihedral angles and secondary structures.

Figure 2 illustrates the ROC curves for turn/non-turn prediction and *β*-turn type prediction before and after using predicted secondary structures and dihedral angles on the GR426 dataset. The ROC curves on datasets FA547 and FA823 are shown in additional file 1. The corresponding areas under the curves were calculated and are presented in tables 5 and 7 for turn/non-turn prediction and *β*-turn type prediction, respectively. The improvement in the results highlights the utility of predicted dihedral angles and secondary structure in both turn/non-turn and *β*-turn type prediction methods.

### DEBT web-server

**Figure 3:**
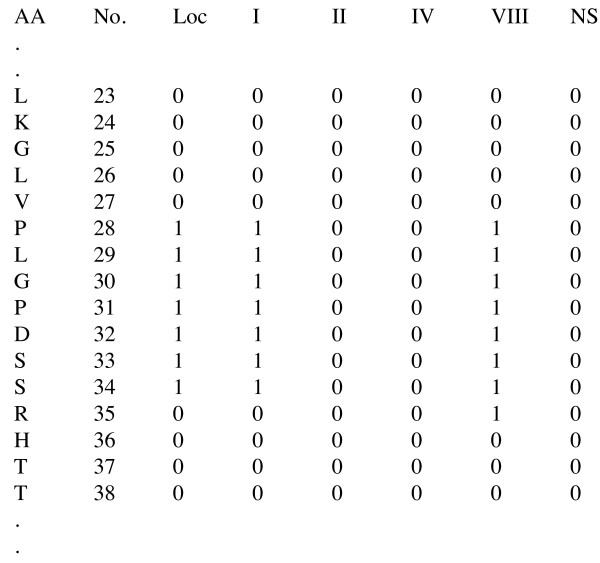
**An example of an output file produced in DEBT web-server**. The first and second columns show the one-letter code and the number of the amino acids, respectively. Column three shows the prediction value of the turn/non-turn prediction and columns four to eight show the prediction values for *β*-types I, II, IV, VIII and NS, respectively. A prediction value can be "1" if the corresponding residue is predicted in *β*-turn/*β*-turn type and "0" otherwise.

Our method is freely available online at http://comp.chem.nottingham.ac.uk/debt/. The web-server was trained using a large training set of 1296 protein chains with at least one *β*-turn to improve the performance of the method. It is written in Perl using a CGI interface. The user can either cut and paste the amino acid sequence or upload a FASTA file. Additionally, multiple FASTA files can be uploaded in an archive. Initially, DEBT firstly runs the PSI-BLAST algorithm [46] to construct the PSSMs and DISSPred [5] to predict the secondary structures and the dihedral angles. Subsequently, the results are merged to create the input file for six SVM classifiers. The output file, shown in figure 3, contains the number and the one-letter abbreviation of the amino acids with six binary prediction values: one for turn/non-turn prediction and five for the *β*-turn types. The prediction value can be "1" if the corresponding residues is predicted in a *β*-turn/*β*-turn type and "0" otherwise. Moreover, the user can ask for DISSPred's results to be attached in the output file, which makes DEBT not only a *β*-turn prediction server, but also a three-state secondary structure prediction and a seven-state dihedral prediction interface. The output file, together with the log files, are sent to the user by e-mail, or can be downloaded, after the calculations are completed. The combination of DISSPred's iterative process with the training on a large dataset makes DEBT web-server slightly slower, but more accurate, than other *β*-turn prediction servers.

## Conclusions

In this article, we presented a method that predicts the location of *β*-turns and their types in a protein chain. Our method uses predicted dihedral angles from DISSPred [5] to enhance the predictions. Moreover, we improved the predictive performance by using a local window of predicted secondary structures and dihedral angles, rather than the predictions for one individual residue. The MCC of 0.48, achieved for turn/non-turn prediction on a set of 426 non-redundant proteins, shows that DEBT is more accurate than other *β*-turn prediction methods. Moreover, we report the highest MCCs of 0.49 and 0.48 on two larger datasets of 547 and 823 non-redundant protein chains. Additionally, the dihedrally enhanced prediction for *β*-turn types is more accurate than other methods. We report DEBT's prediction on three datasets with achieved MCCs up to 0.39, 0.33, 0.27, 0.14 and 0.38 for *β*-turn types I, II, IV, VIII and NS, respectively. The prediction of *β*-turn types has limitations derived from the observation that identical tetrapeptides may form different *β*-turn types. In fact, around 15% of all tetrapeptides that form *β*-turns in datasets GR426 and FA547 appear in multiple *β*-turn types. This number is close to 18% in the FA823 dataset. A detailed analysis of the fundamental limitation of *β*-turn prediction is a challenging future focus. In spite of the limitations, the performance might be improved further by applying techniques introduced by other studies, such as feature selection techniques [34], or by using predicted secondary structures and dihedral angles from multiple predictors. Predicted *β*-turns can be used to improve secondary structure prediction [59] and we are currently exploring this.

## Authors' contributions

PK carried out the experiments and wrote the manuscript. JDH conceived the study and assisted in writing the manuscript. Both authors read and approved the final manuscript for publication.

## Supplementary Material

Additional file 1 **ROC curves for datasets FA547 and FA823**. ROC curves for the predictions on datasets FA547 and FA823, before and after using predicted dihedral angles and secondary structures. Dashed curves correspond to the PSSM-only prediction, while solid curves correspond to the prediction after aumenting the input vector with predicted dihedral angles and secondary structures. Click here for file
