# Supplementary material for: Predicting β-turns and their types using predicted backbone dihedral angles and secondary structures
Source: BMC Bioinformatics. 2010 Jul 31;11:407. doi: 10.1186/1471-2105-11-407 (PMC2920885; doi:10.1186/1471-2105-11-407)

Additional File 1

Dataset FA547

a) turn/non-turn

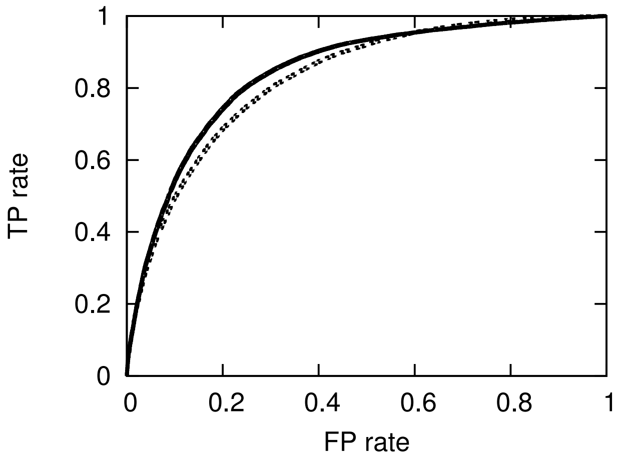

b) type I

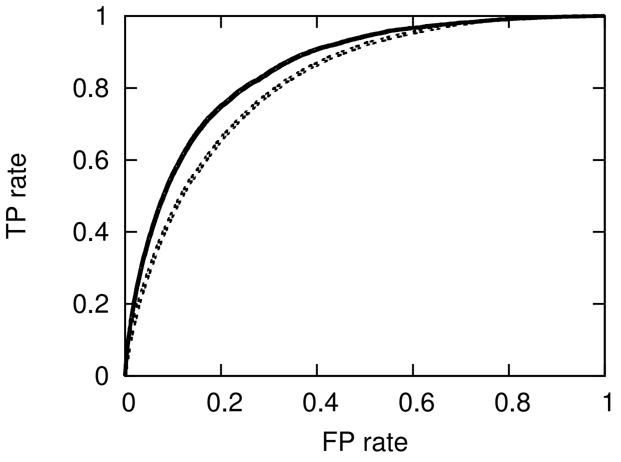

c) type II

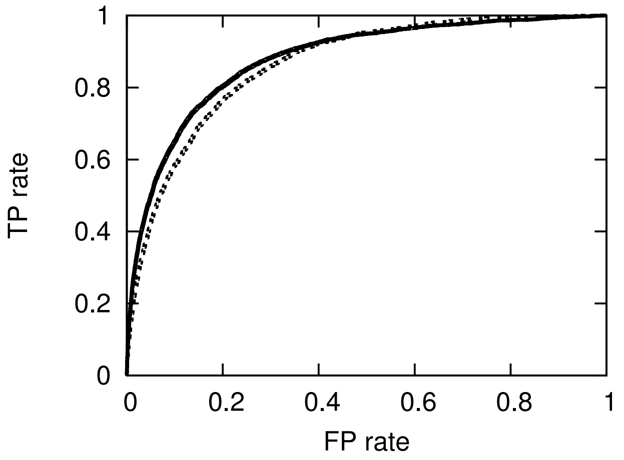

d) type IV

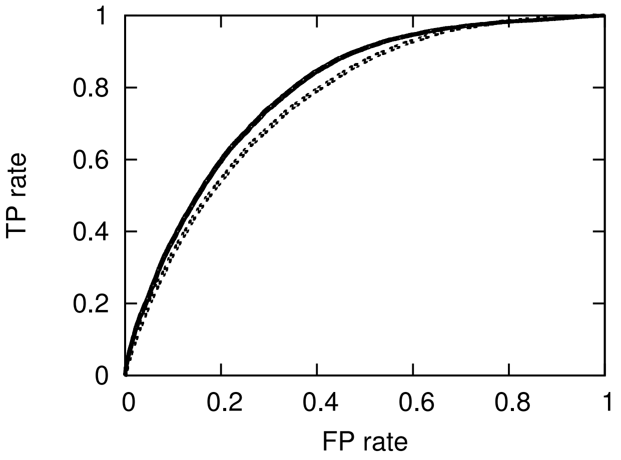

e) type VIII

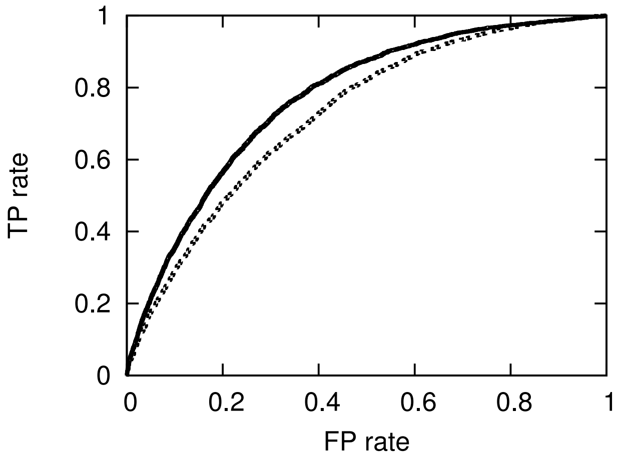

f) type NS

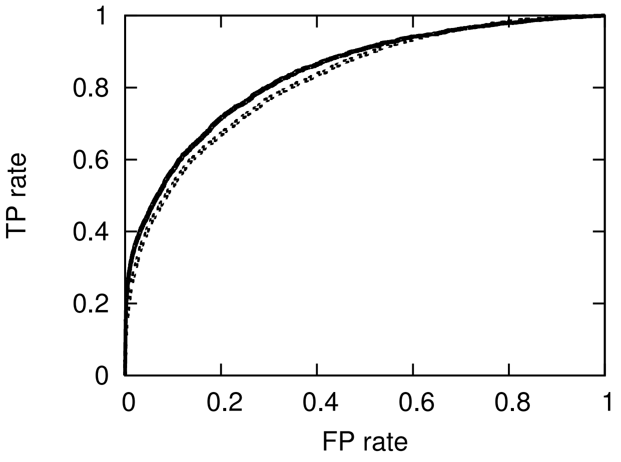

Dataset FA823

a) turn/non-turn

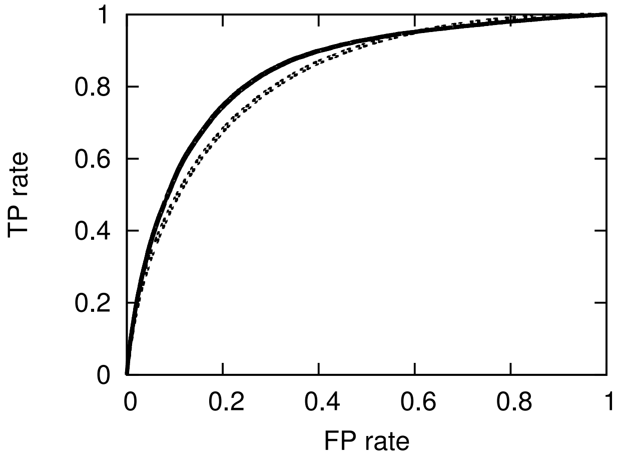

b) type I

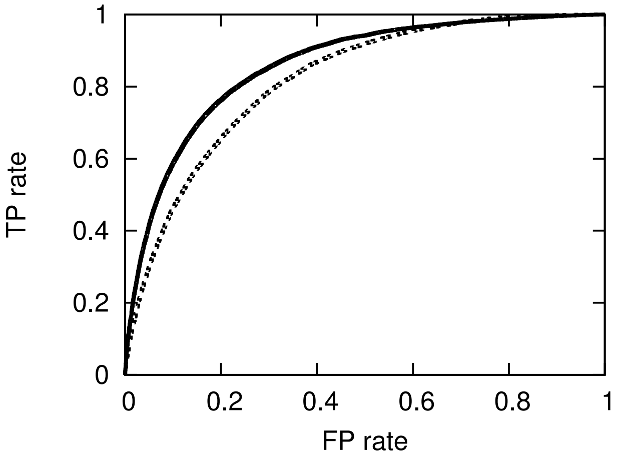

c) type II

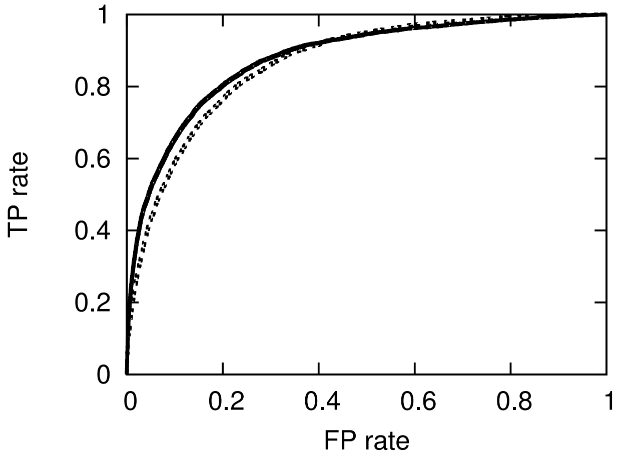

d) type IV

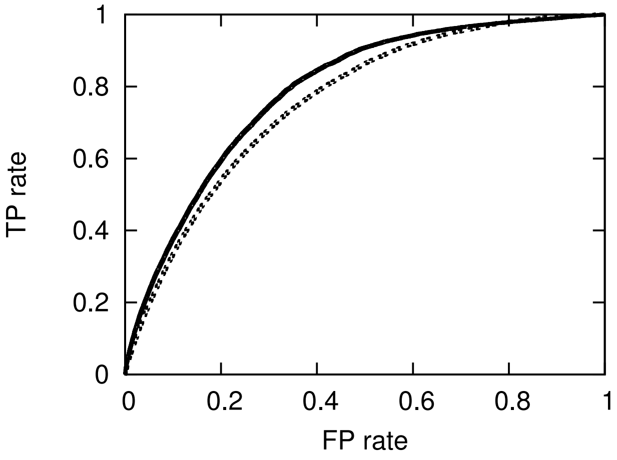

e) type VIII

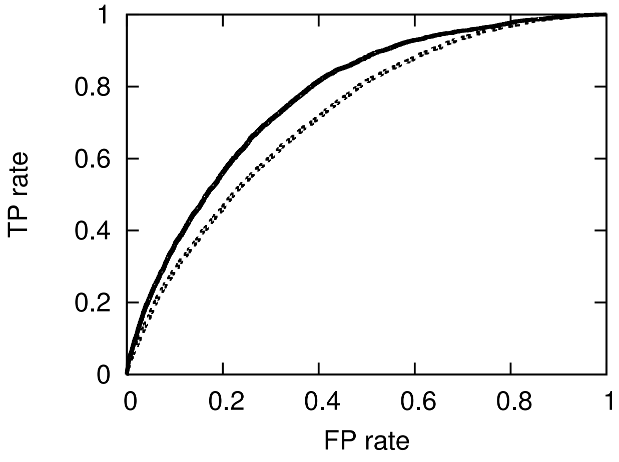

f) type NS

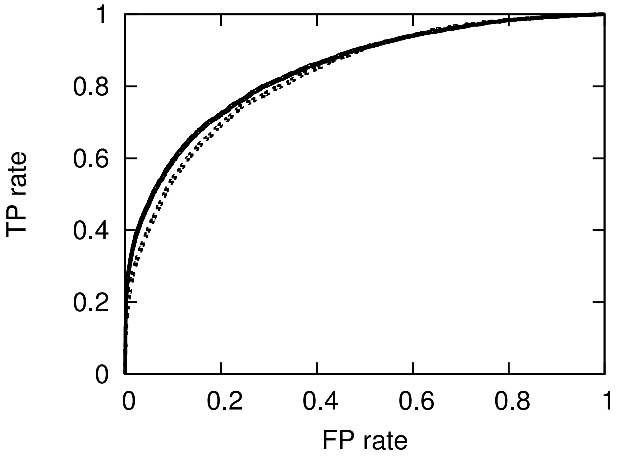

Supplement: Additional file 1 — ROC curves for datasets FA547 and FA823. ROC curves for the predictions on datasets FA547 and FA823, before and after using predicted dihedral angles and secondary structures. Dashed curves correspond to the PSSM-only prediction, while solid curves correspond to the prediction after aumenting the input vector with predicted dihedral angles and secondary structures. [file 1471-2105-11-407-S1.PDF]
